# Value of early change of serum C reactive protein combined to modified Alvarado score in the diagnosis of acute appendicitis

**DOI:** 10.1186/s12873-018-0166-5

**Published:** 2018-05-24

**Authors:** Mohamed Amine Msolli, Kaouther Beltaief, Wahid Bouida, Nahla Jerbi, Mohamed Habib Grissa, Hamdi Boubaker, Riadh Boukef, Semir Nouira

**Affiliations:** 1 Emergency Department, FattoumaBourguiba University Hospital Monastir, 5000 Monastir, Tunisia; 2 Emergency Department, Mahdia University Hospital, 5100 Mahdia, Tunisia; 3 Emergency Department, Sahloul University Hospital, 4011 Sousse, Tunisia; 4 Research Laboratory (LR12SP18), University of Monastir, 5000 Monastir, Tunisia

**Keywords:** C-reactive protein, Acute appendicitis, Alvarado score

## Abstract

**Background:**

The aim of this study is to test the diagnostic value of baseline and early change of C-reactive protein (CRP) concentrations, evaluated separately or in combination with the modified Alvarado score (MAS), in patients with clinically suspected acute appendicitis.

**Methods:**

This is a prospective observational study including all patients presenting to the emergency department with an equivocal diagnosis of acute appendicitis. After inclusion, clinical and demographic data are recorded and blood samples were taken at baseline and 3 h after for serum CRP measurements (3 h CRP). The MAS is also calculated for all patients. The ultimate diagnosis of appendicitis was based on the histologic findings of the excised appendix in operated patients and clinical follow-up in emergency department discharged patients. Diagnostic accuracy of admission CRP, early change of CRP 3 h CRP minus admission CRP, MAS and the combination of these parameters was expressed by sensitivity, specificity, positive predictive value, negative predictive value and area under receiver operating characteristics curve.

**Results:**

Five hundred patients were included from January 2010 to December 2013. Overall, 387 patients were operated the negative appendectomy rate was 8,3%. CRP concentrations were higher in patients with acute appendicitis. However, the diagnostic value of admission CRP, delta CRP and MAS was moderate with area under ROC curve respectively equal to 0.63, 0.53 and 0.6. Combining admission CRP and delta CRP values to MAS did not result in a better performance. The area under ROC curve did not exceed 0.7 with the different combinations.

**Conclusion:**

Early change of CRP has a moderate diagnostic value in patients with clinically suspected acute appendicitis. Combining CRP values to MAS did not improve diagnostic accuracy.

## Background

Acute appendicitis is the most common surgical emergency and the most common source of community-acquired intra-abdominal infections [1]. Clinical diagnosis of acute appendicitis is still difficult. It has been estimated that the diagnostic accuracy of acute appendicitis is between 70 and 85% [2], and that up to 50% of patients hospitalized for possible appendicitis have normal appendices [3]. Misdiagnosing acute appendicitis is responsible of two types of outcomes: in one hand, a delay to surgical treatment can that lead to perforation and peritonitis in up to 15% of the cases [4] and, in the other hand, negative appendectomy which is associated with post-operative complications such as wound infection and adhesions [3]. Several approaches have been introduced to improve the diagnostic accuracy of acute appendicitis and therefore to reduce complications. Imaging techniques, especially abdominal ultrasonography [5] and CT scans [6], have been shown to be particularly accurate with a sensitivity and specificity overcoming 90% [7]. However, doubts have been raised about their usefulness in patients with high clinical probability of acute appendicitis associated with the need for qualified staff and medical facilities contributing to the increase of healthcare costs [8, 9]. Diagnostic scoring systems have been developed in an attempt to improve the diagnostic accuracy of acute appendicitis. The most prominent of those scores is that developed by Alvarado [10, 11]. The modified Alvarado score (MAS) is a more simplified and practical version of the original one and has been widely accepted after it was successfully tested in different studies [12, 13]. Modified Alvarado score is based on three symptoms, three signs, and one laboratory investigation and ranged from 1 to 9 (Table 1).

**Table 1:** Modified Alvarado Score

Symptoms	Score
Migratory right iliac fossa pain	1
Nausea/Vomiting	1
Anorexia	1
Signs	
Tenderness in right iliac fossa	2
Rebound tenderness in right iliac fossa	1
Elevated temperature	1
Laboratory findings	
Leukocytosis	2
Total	9

However, prospective studies have suggested that Alvarado score and MAS alone are inadequate as a diagnostic test for appendicitis [12–14]. During the evaluation of patients with possible appendicitis in the emergency department (ED), repeated physical examination of the abdomen may provide further information to help decision making. Repeated laboratory tests were also proposed to this issue without proven benefit [15].

C-reactive protein (CRP) is an acute phase protein that is often relied-on by many surgeons as a diagnostic marker of acute appendicitis [16]. Actually, there is no strong evidence supporting its use in the diagnosis of acute appendicitis and related clinical data are controversial [2]. The purpose of this study was to investigate the diagnostic performance of initial serum CRP measurement at admission and its early variation, evaluated both separately and in combination to the MAS in patients presenting to the ED with clinically suspected appendicitis.

## Methods

This was a prospective observational study. We prospectively included all patients aged more than 10 years and admitted to our ED for clinically suspected acute appendicitis. The clinical suspicion of appendicitis was made based on the presence of direct tenderness in the right lower quadrant, percussion and rebound tenderness, pyrexia, anorexia, nausea and vomiting. Patients using warfarin or heparin, pregnant women, and patients using antibiotics during the study period were excluded. All patients were initially evaluated by the ED physician and demographic, clinical and biological findings were recorded on a specific data form. Blood samples were obtained immediately after admission and analyzed for while blood cell (WBC) count and admission CRP concentrations. Three hours after, a second CRP measurement (3 h CRP) was carried out in patients with an equivocal diagnosis of acute appendicitis. The CRP concentration was measured by immune turbidimetry (Beckman Collin, CA). The normal range of CRP concentrations, in our hospital, is between 0 and 6 mg/ml and concentrations > 6 mg/L were considered abnormal. At the term of this evaluation, the MAS was calculated for all the included patients. A score above 4 was considered highly suggestive of acute appendicitis. Surgical decision was made at the discretion of senior surgeons based on medical history, clinical examination and initial blood cell count only. All appendectomies were performed by conventional methods and surgeons were blinded about the CRP values. In patients for whom the surgical treatment was decided within the 2 weeks of ED presentation, the ultimate diagnosis of appendicitis was based on histologic examination of the excised appendix. Appendicitis was defined as ulcerative, suppurative, phlegmonous, gangrenous or perforated appendicitis. Absence of appendicitis was considered if, in home discharged patients, initial symptoms has subsisted within the 2-week follow-up at the outpatient clinic; and, in operated patients, a normal appendix was found at the histopathological examination. All patients gave their written informed consent to participate in the study which was approved by our institutional ethics committee.

### Statistical analysis

Data were presented as mean ± SD (standard deviation) or as percentages. The clinical data were compared using the student-t test for the continuous variables and the Chi-square test for categorical variables. The admission CRP and delta CRP (3 h CRP – admission CRP) diagnostic performance was evaluated alone and in combination to the MAS by the calculation of the sensitivity, specificity, positive and negative predicted values for each category and the calculation of the area under curve (AUC) of the receiver operating characteristic (ROC) presentation. The closer AUC is to 1, the better is the overall performance of the test. An AUC =0.5 means that the test is not better than a random one. All *p* values < 0.05 were considered statistically significant.

## Results

**Fig. 1:**
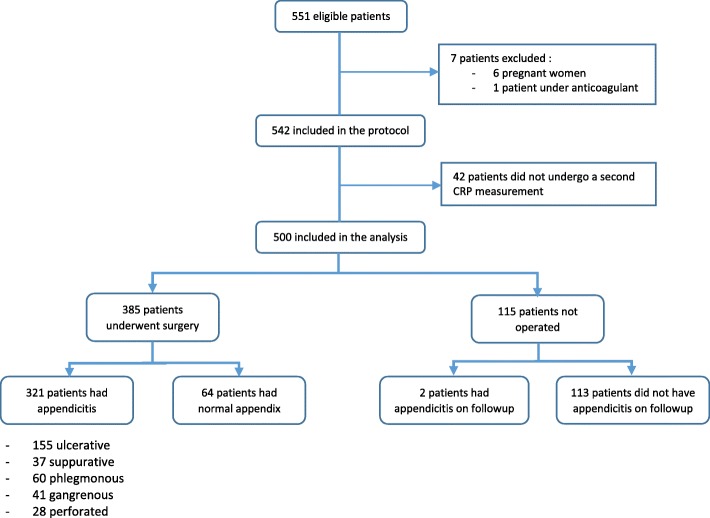
Patients flow chart

During the study period, 551 patients were eligible from them 542 patients were admitted. 42 patients did not undergo a second CRP measurement and a total of 500 patients with clinically suspected appendicitis were included in the study. The patients mean age was 28 ± 3 years with extremes ranging from 8 to 85. Young subjects aged under 30-year-old and males were the most frequent (Table 2). Most of the patients (*n* = 385) underwent appendectomy within the first 24 h of ED admission. In the operated group, negative appendectomy rate was 8,3% (64 cases). Histologic findings included 155 (40%) ulcerative, 37 (9.6%) suppurative, 60 (15.5%) phlegmonous, 41 (10.6%) gangrenous and 28 (7.2%) perforated appendices. Only two patients (1.7%) in the non-operated group had been diagnosed with acute appendicitis during their 2-week follow-up (Fig. 1). Mean WBC count, mean level of admission CRP and 3 h CRP and mean delta CRP between patients with and without appendicitis are shown in Table 3. In patients with acute appendicitis mean WBC and CRP measurements were significantly higher than in the patients with normal appendix (both *p* < 0.01). The delta CRP was positive in 73% patients with appendicitis and in 46% patients with normal appendix; the difference was significant (*P* < 0.001).

**Table 2:** Baseline characteristics of the patients enrolled in the study

	Appendicitis *n* = 323 (64.6%)	No appendicitis *n* = 177 (34.4%)	*p*
Sex (male: female)	181: 142	74: 103	0.002
Age (y); mean (SD)	28 (13)	26 (12)	0.17
Pain duration n (%)			0.12
< 6 h	24 (7,4)	21 (11,8)	
6–12 h	72 (22,2)	43 (24,2)	
12–24 h	138 (42,7)	68 (38,4)	
24–48 h	69 (21,3)	37 (20,9)	
> 48 h	20 (6,2)	8 (4,5)	
Associated symptoms, n (%)			
Nausea and vomiting	145 (44,8)	69 (38,9)	0.20
Anorexia	29 (8,9)	15 (8,4)	0.84
Temperature at presentation, n (%)			0.016
Afebrile	12 (3,7)	13 (7,3)	
37–37.9	187 (57,8)	118 (66,6)	
38–38.9	112 (34,6)	38 (21,4)	
> 39 °C	12 (3,7)	8 (4,5)	
Physical examination, n (%)			
RLQ tenderness	268 (82,9)	132 (74,5)	0.025
Rebound tenderness	227 (70,2)	91 (51,4)	0.036
RLQ defense	74 (22,9)	21 (11,8)	0.003
Modified Alvarado score, n (%)			< 0.001
≤ 4	142 (44)	115 (65)	0.014
s> 4	181 (56)	62 (35)	

RLQ: right lower quadrant of the abdomen

**Table 3:** Comparison of C-reactive protein values and modified Alvarado score between patients with and without appendicitis

	Appendicitis, n (%) *n* = 323 (64.6%)	No appendicitis, n (%) *n* = 177 (35.4%)	*p*
White blood cell count, 1000/mm^3^	14.6 (5.9)	12.1 (4.1)	0.002
Admission CRP mg/L, mean (SD)	40.4 (30.5)	20.3 (42.9)	< 0.001
Admission CRP mg/L, median (IQ range)	16.3 (5.4–60)	4,8 (1.1–14.9)	
3 h CRP mg/L, mean (SD)	48.4 (31.9)	21.9 (23.5)	< 0.001
3 h CRP mg/L, median (IQ range)	26.9 (8.3–77.9)	7.9 (1.6–24.3)	
Delta CRP mg/L, mean (SD)	+ 9.7 (23.3)	+ 1.8 (28.5)	0.001
Delta CRP mg/L, median (IQ range)	3.7 (0.5–11.9)	1.1 (0.06–4.6)	
Modified Alvarado score, mean (SD)	4.5 (1.5)	3.8 (1.6)	< 0.001

CRP: C-reactive protein

Delta CRP: admission CRP minus 3 h CRP

**Fig. 2:**
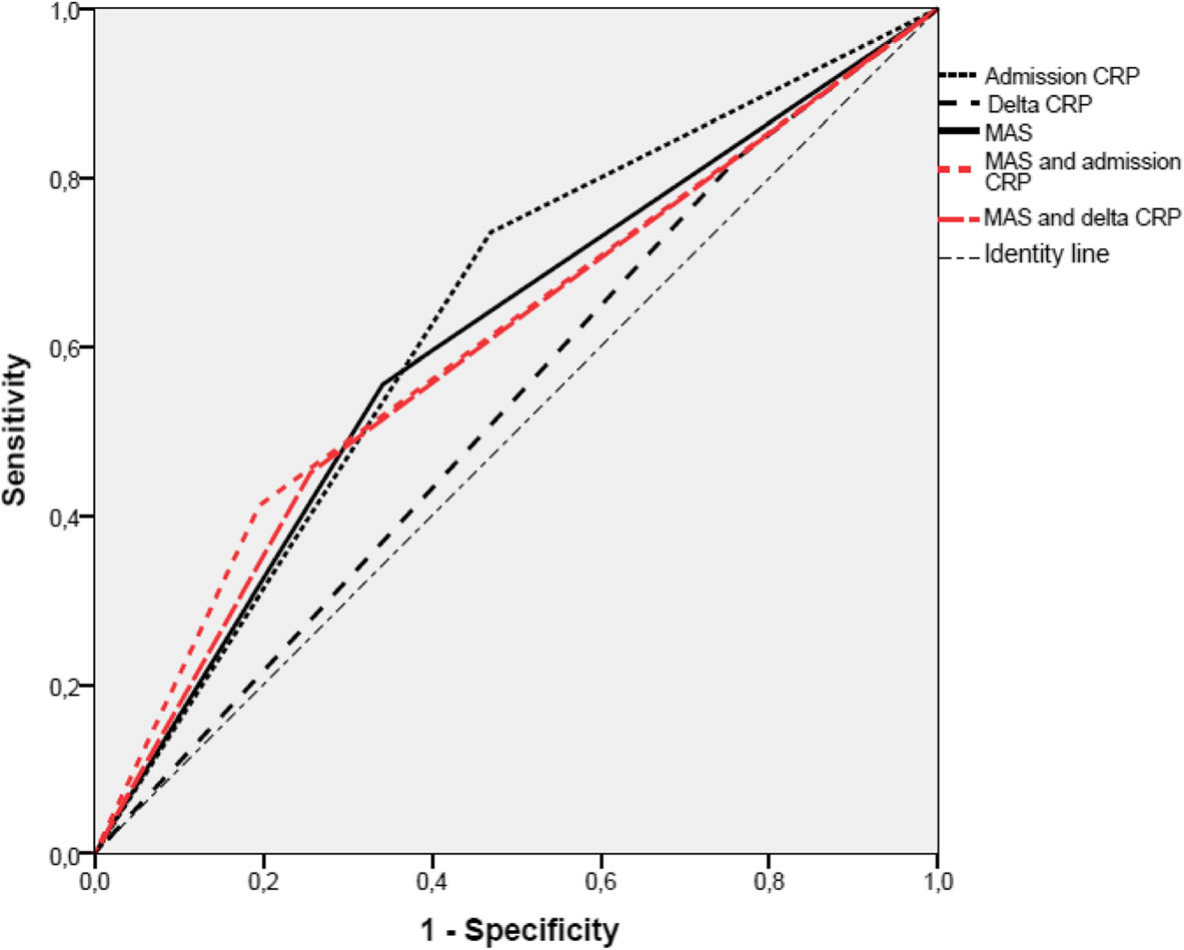
Area under ROC curve of admission CRP (0.63), delta CRP (0.53), modified Alvarado score (0.6), MAS combined to admission CRP (0.6) and MAS combined to delta CRP (0.59). Delta CRP = admission CRP minus 3 h CRP

The diagnostic performance of both CRP and delta CRP in predicting acute appendicitis were further analyzed by using the ROC curves (Fig. 2). The AUC for admission CRP and 3 h CRP was respectively 0.63 and 0.65. However, for delta CRP it was only 0.53. The performance (sensitivity, specificity, PPV and NPV) of admission CRP and delta CRP analyzed separately and in combination to the MAS, in the diagnosis of acute appendicitis were shown in Table 4. Both admission CRP and delta CRP had a better sensitivity than MAS alone (73, 84 and 56% respectively). Combining admission CRP or delta CRP to MAS was associated with a reduced sensitivity and a moderate increase of specificity compared to MAS alone. Moreover, applying admission CRP and delta CRP to each of MAS risk category (MAS ≤ 4 versus MAS >  4) did not improve the diagnostic performance (Table 5).

**Table 4:** Performance of C-reactive protein values, MAS and their combinations in the diagnosis of acute appendicitis

	Sensitivity (95% CI)	Specificity (95% CI)	PPV (95% CI)	NPV (95% CI)	LR+ (95% CI)	LR- (95% CI)
MAS > 4 alone	56 (50–61)	64 (57–71)	74 (68–79)	44 (38–51)	1.55 (1.16–2.1)	0.68 (0.54–0.87)
Elevated admission CRP	73 (68–78)	53 (45–60)	74 (69–78)	52 (44–59)	1.55 (1.23–1.95)	0.50 (0.36–0.71)
Positive delta CRP	84 (79–87)	19 (13–26)	66 (61–71)	38 (27–50)	1.03 (0.90–1.17)	0.84 (0.50–1.61)
MAS combined with admission CRP	41 (36–47)	80 (73–85)	79 (72–85)	42 (37–48)	2.05 (1.33–3.13)	0.73 (0.62–0.87)
MAS combined with delta CRP	42 (37–48)	76 (69–82)	76 (69–82)	42 (36–47)	1.75 (1.19–2.66)	0.76 (0.63–0.91)

MAS: Modified Alvarado Score

Delta CRP: admission CRP minus 3 h CRP

**Table 5:** Diagnostic performance of CRP 1 and delta CRP depending on modified Alvarado score risk category

	Low risk (MAS ≤ 4) *n* = 257					High risk (MAS > 4) *n* = 243				
	(95% CI)					(95% CI)				
	Se	Sp	PPV	NPV	AUC	Se	Sp	PPV	NPV	AUC
Elevated admission CRP	73 (65–80)	58 (49–67)	68 (60–76)	63 (53–72)	0.66	74 (67–80)	43 (30–56)	79 (72–85)	36 (25–48)	0.58
Delta CRP > 1 mg/L	83 (76–89)	23 (15–32)	57 (50–64)	53 (38–67)	0.53	81 (74–86)	25 (14–38)	76 (69–82)	30 (17–45)	0.53

Delta CRP: 3 h CRP minus admission CRP

*Se* sensitivity

*Sp* specificity

*PPV* positive predictive value

*NPV* negative predictive value

*AUC* area under receiver operating characteristics curve

## Discussion

The accurate diagnosis of acute appendicitis in the ED remains a challenge for the emergency physician [17]. Good clinical approach with detailed physical examination is keystone in the diagnosis of appendicitis. However, atypical clinical presentations and nonspecific findings are frequent which could delay the diagnosis leading to complications, more often perforation; or carry misdiagnosis and lead to unnecessary surgical interventions. To overcome these difficulties, many diagnostic strategies were developed including short period admission for observation, serial laboratory tests and imaging investigations [18]. Although the utility of repeated laboratory examinations may seem helpful, diagnostic yield of serial tests has not been studied thoroughly enough to be validated in this condition. Also, it is unclear which serum inflammatory markers should be used and how well the early levels changes can function as a discriminator factor in patients with a suspected diagnosis of acute appendicitis [19]. In this prospective study we analyzed 500 patients with suspected appendicitis, and evaluated the utility of early changes in serum CRP concentrations in the diagnosis of acute appendicitis. We demonstrated that both CRP markers were not of significant utility either alone or in combination to MAS in the diagnosis of appendicitis. Our findings contrast with some previous studies suggesting that repeated serum inflammation tests may increase the diagnostic accuracy of acute appendicitis. Recently, Han-ping et al. found that the change between primary and repeated serum inflammatory markers may improve diagnostic accuracy in pediatric appendicitis [20]. ROC analysis showed that a cut-off value of serum CRP more than 4.5 mg/L on day 2, or the increase in CRP above 15.0 mg/L on day 3 were good predictors of acute appendicitis in children. In the same way, Wu HP et al. concluded that the change in the serum parameters could point for simple appendicitis when the increase in CRP is more than 118 mg/L; and that appendicitis could be excluded when the increase in CRP is less than 10 mg/L. For perforated appendicitis, the authors concluded that changes in CRP values was not helpful for the diagnosis [21]. In our study the second measurement of the CRP concentrations was made only 3 h which could explain our negative results. Indeed, several studies have reported that serum CRP increase is delayed 12 to 24 h from the onset of inflammation symptoms [22]. In Practice, repeating serum analysis more than 12 h after ED admission would be very late especially in acute appendicitis context where complications are potentially life-threatening. Our findings highlight the need for other new biomarkers with faster expression and kinetics.

A variable combination of clinical signs has been used together in association with laboratory tests in several scoring systems for evaluating the probability of acute appendicitis. The Alvarado score is the most widely studied score. Because counting the neutrophils as a parameter of the Alvarado score is not routine in many laboratories, a simplified version was proposed (MAS) by omitting the neutrophil count and demonstrated similar diagnostic performance [23]. Some studies have shown enhanced diagnostic potential and utility when scoring systems and inflammatory markers are combined [24]. However, our results did not confirm these findings which suggest that alternative diagnostic approach are needed. Combination of inflammatory markers or the use of novel ones combined to available scores could be of greater promise [25].

As far as we know, this is the largest study to prospectively evaluate the diagnostic value of early variation of CRP concentrations in subjects presenting to the ED with clinically suspected appendicitis. Although the important sample size we acknowledge that this trial has some limitations. First, our study is monocentric, which could lead to a potential selection bias. Moreover, some subjects discharged home with a false diagnosis of non-appendicitis may have presented to other hospitals as for some patients contact information was lacking for follow-up. Finally, in our study, the diagnosis of acute appendicitis was a compromise between patient statement, physical examination and laboratory findings and no imaging tests were performed which could explain the relatively high rate of normal appendices findings at the histological examination.

## Conclusion

Based on our findings, we conclude that CRP levels at admission and its early change 3 h later, in patients with clinically suspected acute appendicitis, has a moderate diagnostic value. Combining values to the MAS added no more diagnostic utility. Other diagnostic approaches should be considered in this context.

## Abbreviations

CRP: C - reactive protein
ED: Emergency Department
MAS: Modified Alvarado Score
